# Persistent organic pollutants and risk of cutaneous malignant melanoma among women

**DOI:** 10.1002/cnr2.1536

**Published:** 2021-08-20

**Authors:** Maryam Darvishian, Parveen Bhatti, Éric Gaudreau, Zenaida Abanto, Charles Choi, Richard P. Gallagher, John J. Spinelli, Tim K. Lee

**Affiliations:** ^1^ Cancer Control Research Program BC Cancer Vancouver British Columbia Canada; ^2^ Centre de Toxicologie du Québec (CTQ) Institut National de Santé Publique du Québec (INSPQ) Québec Canada; ^3^ Faculty of Medicine University of British Columbia Vancouver British Columbia Canada; ^4^ Department of Dermatology and Skin Sciences University of British Columbia Vancouver British Columbia Canada; ^5^ School of Population and Public Health University of British Columbia Vancouver British Columbia Canada

**Keywords:** melanoma, organochlorine pesticides, persistent organic pollutants, polychlorinated biphenyls, skin cancer

## Abstract

**Background:**

Despite the increasing trend of cutaneous malignant melanoma (CMM) incidence in Canada, especially among females, few risk factors other than ultraviolet radiation exposure, have been identified.

**Aim:**

We conducted a case–control study of 406 CMM cases and 181 controls to evaluate the potential impact of body burdens of various persistent organic pollutants on CMM risk.

**Methods:**

Detailed data on potential confounding factors, including lifetime repeated sun exposure and skin reaction to repeated sun exposure, were collected. Gas chromatography tandem mass spectrometry was used to assay plasma levels of 14 polychlorinated biphenyl (PCB) congeners and 11 organochlorine (OC) pesticides among cases and controls.

**Results:**

Statistically significant trends of increased CMM risk were observed with increasing plasma concentrations of multiple PCB congeners, including PCBs 138, 153, 170, 180, 183 and 187. For example, compared to lowest plasma concentration quartile of PCB‐138, the second, third and fourth quartiles were associated with 1.7 (95% CI: 0.9–2.9), 2.3 (95% CI: 1.3–4.1) and 2.4 (95% CI: 1.3–4.5) ‐fold increased risks of CMM, respectively. Similarly, increasing plasma concentrations of several OC pesticides (i.e., β‐HCH, HCB, Mirex, oxychlordane and trans‐Nonachlor) showed statistically significant trends with increased CMM risk. For example, compared to lowest plasma concentration quartile of β‐HCH, the second, third and fourth quartiles were associated with 1.3 (95% CI: 0.7–2.3), 2.1 (95% CI: 1.2–3.7) and 2.3 (95% CI: 1.2–4.4) ‐fold increased risks of CMM, respectively.

**Conclusion:**

Plasma levels of several persistent organic pollutants were highly correlated, suggesting that observed associations were not necessarily independent of each other. Given the highly correlated nature of exposure to PCB and OC analytes, sophisticated analyses that consider complex mixtures should be considered in future studies.

## 1 INTRODUCTION

As in other parts of the world, there has been an increasing trend of cutaneous malignant melanoma (CMM) incidence in Canada. Since 1994, Canadian females have the second highest annual percent change in age‐standardize incidence rate of melanoma over other cancers.^1^, ^2^ Despite the increasing incidence, few risk factors, other than ultraviolet radiation exposure, have been identified.^3^

Polychlorinated biphenyls (PCBs) are a class of persistent organic pollutant (POP; i.e., compounds that bio‐accumulate in the environment, animals and humans and can still be detected in the general population despite their use being banned decades ago) and have been classified as known human carcinogens by the International Agency for Research on Cancer (IARC).^4^, ^5^, ^6^ This was primarily based on evidence of increased melanoma risk among occupationally exposed individuals; however, more recently conducted meta‐analyses, that primarily include highly exposed occupational groups, do not confirm IARC's classification.^7^, ^8^ Few studies of organochlorine (OC) pesticides, another class of POP, and CMM risk have also been conducted and, as with PCBs, the primary focus has been on occupational exposures. For example, in the Agricultural Health Study (AHS), toxaphene exposure, as assessed via questionnaire, was found to be associated with increased CMM risk, but this was not replicated in the AHS after additional years of follow‐up.^9^, ^10^

To assess the potential associations of PCB and OC pesticide exposures with risk of CMM in the general population, we previously conducted a preliminary population‐based case–control study in BC.^4^ Comparing 80 CMM cases to 310 controls, we observed strong associations between plasma levels of various PCB congeners and OC pesticides and risk of CMM.^4^ We have now followed‐up these findings with another study that includes a much larger number of cases. In addition, the study focuses on women given the steeper increases in CMM incidence observed in this group.

## 2 METHODS

### 2.1 Study population

The protocol for this investigation was approved by the Research Ethics Board of the University of British Columbia and the BC Cancer Agency.

**FIGURE 1:**
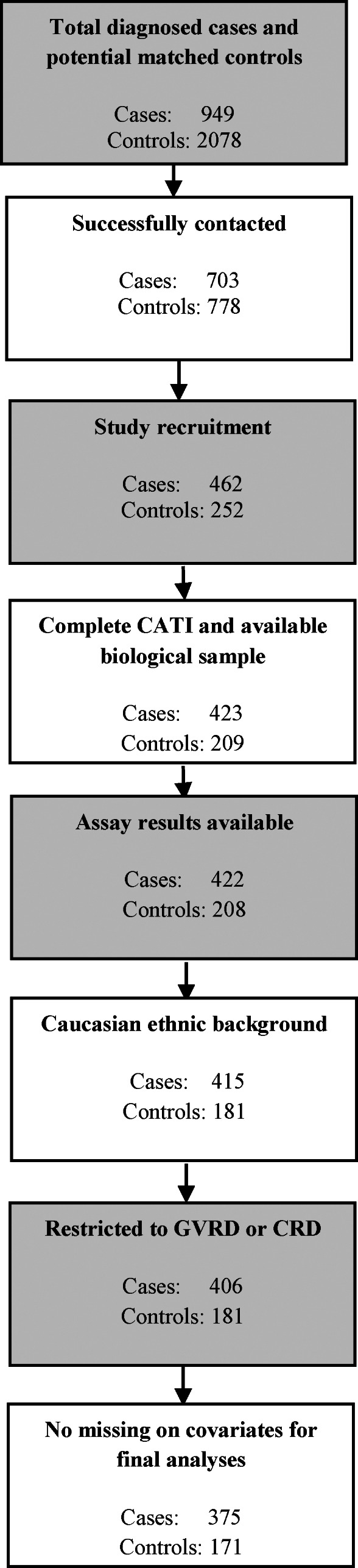
Study flow diagram

Between July 2011 and January 2013, females aged 20–79 with CMM, were identified through the population‐based BC Cancer Registry. A total of 703 cases were successfully contacted by telephone (up to three contact attempts were made), with 241 (34%) refusing to participate, leaving 462 (66%) that consented to participate in the study (Figure 1).

From December 2012 to May 2014, 2078 cancer‐free controls, frequency matched to cases on age and residential district, were randomly selected from the consolidation file of the British Columbia Ministry of Health, which contains identifying information on all participants in the population‐based health insurance plan. A total of 778 were successfully contacted by telephone, with 526 (67%) refusing to participate, leaving 252 (32%) that consented to participate in the study (Figure 1).

### 2.2 Data and blood sample collection

All study participants were asked to complete a computer‐assisted telephone interview (CATI) to provide information on sun exposure, medical history, lifestyle factors, family history of melanoma, occupational history, and residential history. Participants were also asked to provide a whole blood sample, which was collected in EDTA tubes at a community laboratory and shipped, on ice, to BC Cancer within 24 h of collection for processing. A volume of 2 ml of plasma was separated from whole blood by centrifugation, transferred to vials with a Teflon stopper and frozen at −80°C.

### 2.3 PCB and OC pesticide assays

De‐identified plasma samples were shipped in random batches of cases and controls to the Centre de Toxicologie du Québec (CTQ), Institut National de Santé Publique du Québec (INSPQ) for assay. Laboratory staff were blinded to case–control status. Fourteen PCB congeners (IUPAC nos. 28, 52, 99, 101, 105, 118, 128, 138, 153, 156, 170, 180, 183 and 187) and 11 OC pesticides (aldrin, β‐hexachlorocyclohexane (β‐HCH), α‐chlordane, γ‐chlordane, cis‐Nonachlor, trans‐Nonachlor, p,p'‐DDT, p,p'‐DDE, hexachlorobenzene (HCB), mirex, and oxychlordane), which were selected based on observations in the preliminary study,^4^, ^11^ were measured using the gas chromatography‐ mass spectrometry (GC–MS) where the analytical method was described in Fisher et al.^12^ The limit of detection (LOD) varied from 0.01 to 0.3 μg/L for PCBs and varied from 0.005 to 0.09 μg/L for OCs (Table 1).Concentrations below the LOD were assigned a value of the LOD divided by √2.^4^, ^13^

**TABLE 1:** Number of samples measured above limit of detection

PCB congeners/pesticide analytes	LOD (μg/L)	Cases (*n* = 406)		Controls (*n* = 181)		
		*N*	%	*N*	%	Median^b^(μg/kg of lipids)
*PCB congeners*						
28^a^	0.05	13	3.2%	9	5.0%	‐
52^a^	0.3	0	0.0%	0	0.0%	‐
99	0.03	104	25.6%	48	26.5%	‐
101^a^	0.03	7	1.7%	4	2.2%	‐
105	0.01	320	78.8%	153	84.5%	0.8
118	0.01	405	99.8%	181	100.0%	4.4
128	0.01	37	9.1%	15	8.3%	‐
138	0.01	406	100.0%	181	100.0%	9.0
153	0.01	406	100.0%	181	100.0%	21.3
156	0.01	406	100.0%	181	100.0%	2.9
170	0.01	406	100.0%	181	100.0%	6.4
180	0.01	406	100.0%	181	100.0%	1.9
183	0.01	401	98.8%	179	98.9%	1.5
187	0.01	406	100.0%	181	100.0%	4.8
*Pesticide analytes*						
Alpha‐chlordane^a^	0.01	2	0.5%	0	0.0%	‐
Aldrin^a^	0.01	0	0.0%	0	0.0%	‐
β‐HCH	0.01	392	96.6%	179	98.9%	5.4
cis‐Nonachlor	0.005	400	98.5%	178	98.3%	0.8
Gamma‐chlordane^a^	0.005	4	1.0%	0	0.0%	‐
p,p'‐DDE	0.09	406	100.0%	181	100.0%	102.0
p,p'‐DDT^a^	0.05	47	11.6%	42	23.2%	‐
HCB	0.04	406	100.0%	181	100.0%	9.5
Mirex	0.01	322	79.3%	133	73.5%	‐
Oxychlordane	0.005	396	97.5%	172	95.0%	4.7
trans‐Nonachlor	0.01	406	100.0%	181	100.0%	6.9

^a^ Excluded from further analysis due to ≤20% values above the detection limit.

^b^ Median lipid‐adjusted concentration among controls of analytes with >80% of measurements above the limit of detection.

Using enzymatic methods, free cholesterol (FC), total cholesterol (TC), triglycerides (TG), and phospholipids (PL) were measured in each plasma sample.^4^ Total lipid concentration was calculated using the Akins summation formula.^4^, ^12^, ^14^ Lipid‐adjusted concentrations (μg/kg of lipids) were calculated by dividing the whole‐weight measurements of each analyte by the total lipid concentration.^4^, ^14^

Blood sample collection and processing used the same methods for both melanoma cases and NHL controls as previously described. All organochlorine assays were performed at the Centre de Toxicologie in Quebec, Canada; assays for the con‐trols between 2002 and 2005, and for CMM cases in 2008.

### 2.4 Statistical analysis

For those analytes with greater than 80% of samples above the LOD, Spearman rank correlations between the various analytes were examined. Analytes with ≤20% observations above the LOD were excluded from further analyses (aldrin, α‐chlordane, γ‐chlordane, DDT and PCB congeners nos. 28, 52, 101 and 128). For PCB 99, which only had ~26% of observations above the LOD, concentrations were categorized as detectable or non‐detectable. For mirex, in addition to a non‐detectable category, the >70% of detectable values were dichotomized based on the distribution among controls. All other analytes were categorized into quartiles according to their distribution among controls. The lowest quartile was used as the reference category. Total PCB level, total dioxin‐like PCB level (PCB nos. 118 and 156) and total non‐dioxin‐like PCB level (PCB nos. 138, 153, 170, 180, 183, and 187) were computed by summing the individual lipid‐adjusted serum concentrations of each PCB congener.^4^, ^15^ These summary PCB levels were also categorized into quartiles according to distributions among controls.

Unconditional logistic regression was used to estimate odds ratios (OR) and 95% confidence intervals (95% CI) for risk of CMM in association with each PCB congener and pesticide individually as well as the various summary PCB measures. Tests for trends across the quartiles were calculated by creating a continuous trend variable equal to the median analyte levels within each category for controls for each analyte and aggregate metric. We evaluated potential confounders in our analyses including age, education, hair color, skin color, moles, skin reaction to first sun exposure without sunscreen, skin reaction to repeated sun exposure without sunscreen, total sun exposure during warm months, sun exposure during vacation in warm regions, and recreational sun exposure, the instrument used to evaluate sunlight exposure variables has been well validated and used successfully in a number of previous melanoma studies in populations in multiple countries.^16^, ^17^, ^18^ Age was examined in four categories (20–49, 50–59, 60–69 and 70–79). In separate models for each analyte, if inclusion of a covariate changed the OR estimate for that analyte by more than 5%, the covariate was included in the final statistical model.

All analyses were performed using SPSS for Windows, version 24.0.

## 3 RESULTS

As shown in Figure 1, 39 cases and 43 controls were excluded due to missing samples and incomplete CATI. After further exclusion of participants with missing assay results (1 case and 1 control), non‐Caucasian participants (7 cases and 27 controls) and those residing outside the Greater Vancouver Regional District and Capital Regional District (9 cases), 406 cases and 181 controls were included in the analyses (as shown in Tables 1 and 3). For multivariate analyses (Tables 4 and 5), only 375 cases and 171 controls were used due to missing covariate data.

Information on the number of samples measured above the LOD for each analyte is provided in Table 1. Statistically significant correlations between plasma levels of various PCB and pesticide analytes were observed (*p* < .001). The strongest correlations were observed between PCB congeners 170, 180, and 187 (*r* > 0.90) and between oxychlordane and trans‐Nonachlor (*r* = 0.939) (Table 2).

**TABLE 2:** Spearman's rank correlation between individual organochlorine pesticide analytes and PCB congeners

	β‐HCH	*cis*‐Nonachlor	*p,p'*‐DDE	HCB	Oxychlordane	*trans*‐Nonachlor	PCB no. 105	PCB no. 118	PCB no. 138	PCB no. 153	PCB no. 156	PCB no. 170	PCB no. 180	PCB no. 183	PCB no. 187
β‐HCH	_	0.7	0.6	0.7	0.7	0.7	0.6	0.7	0.7	0.7	0.6	0.6	0.6	0.7	0.6
*cis*‐Nonachlor		_	0.6	0.7	0.8	0.9	0.7	0.7	0.7	0.7	0.7	0.6	0.6	0.7	0.7
*p,p'*‐DDE			_	0.6	0.7	0.6	0.6	0.7	0.8	0.7	0.5	0.5	0.5	0.8	0.6
HCB				_	0.8	0.7	0.6	0.7	0.7	0.7	0.7	0.6	0.6	0.7	0.7
Oxychlordane					_	0.9	0.6	0.7	0.7	0.8	0.8	0.7	0.7	0.7	0.7
*trans*‐Nonachlor						_	0.6	0.7	0.7	0.7	0.7	0.7	0.7	0.7	0.7
PCB no. 105							_	0.9	0.7	0.6	0.5	0.5	0.5	0.6	0.5
PCB no. 118								_	0.8	0.8	0.7	0.6	0.6	0.7	0.7
PCB no. 138									_	0.9	0.8	0.8	0.7	0.9	0.8
PCB no. 153										_	0.9	0.9	0.9	0.9	0.9
PCB no. 156											_	0.9	0.9	0.8	0.9
PCB no. 170												_	1.0	0.8	0.9
PCB no. 180													_	0.8	0.9
PCB no. 183														_	0.9
PCB no. 187															_

In general, cases were slightly younger than controls (mean age: 55 vs. 60) and were less educated (bachelor's degree or higher: cases 34.0 vs. 40.9%; Table 3). On average, cases reported a 1.1 pound increase in weight in the time between study entry and 2 years before study entry (i.e., before diagnosis and treatment), while controls reported, on average, a 0.1 pound increase in weight during this time (Table 3). Additionally, compared to controls, a higher proportion of cases had light hair color, fair skin color, and reported many moles. These differences reflect well known differences seen in virtually all studies of melanoma and UV exposure.

**TABLE 3:** Characteristics of CMM cases and controls

Characteristics	Case *N* = 406		Control *N* = 181	
	N^a^	%	N^a^	%
Mean age at diagnosis (*SD*)	55 (13)	‐	60 (12)	‐
Mean age at entry in study (*SD*)	58 (13)	‐	60 (12)	‐
*Age group*				
20–49	129	31.8%	31	17.1%
50–59	110	27.1%	49	27.1%
60–69	111	27.3%	65	35.9%
70–79	56	13.8%	36	19.9%
Mean weight (lbs) at entry in study (*SD*)	155 (32)		151 (28)	
Mean weight (lbs) 2 years before study entry (*SD*)	154 (32)		150 (27)	
*Education*				
Lower than or completed secondary	153	37.7%	59	32.6%
Trade or College certificate	115	28.3%	48	26.5%
Bachelor's degree or higher	138	34.0%	74	40.9%
*Eye color*				
Dark	64	15.8%	39	21.5%
Light	342	84.2%	142	78.5%
*Natural hair color*				
Dark	122	30.0%	78	43.1%
Light	284	70.0%	103	56.9%
*Skin color*				
Dark	76	18.7%	59	32.6%
Fair	328	80.8%	122	67.4%
Missing	2	0.5%	0	0
*Degree of freckling*				
None	175	43.1%	91	50.3%
Few	139	34.2%	56	30.9%
Many	92	22.7%	33	18.2%
Missing	0	0	1	0.6%
*Moles*		0		0
None	97	23.9%	59	32.6%
Few	230	56.7%	101	55.8%
Many	79	19.5%	20	11.0%
Missing	0	0	1	0.6%
*Employment status*				
Full time	163	40.1%	59	32.6%
Part‐time	62	15.3%	27	14.9%
Others	181	44.6%	95	52.5%
*Marital Status*				
Married	286	70.4%	119	65.7%
Single/divorced/widowed	120	29.6%	61	33.7%
Missing	0	0	1	0.6%
*Lifetime number of sunburns*				
<=5	120	29.6%	61	33.7%
>5	271	66.7%	120	66.3%
Missing	15	3.7%	0	0
*Sunburn in childhood*				
<=5	103	25.4%	63	34.8%
>5	11	2.7%	4	2.2%
DK	62	15.3%	19	10.5%
Missing	230	56.7%	95	52.5%
*Skin reaction when first exposed to midday sun in summer without protection*				
Tan with no sunburn/no change in skin color	257	63.3%	26	14.4%
Mild sunburn followed by some suntan	102	25.1%	52	28.7%
Sunburn with or without blisters	40	9.9%	102	56.4%
Missing	7	1.7%	1	0.6%
*Skin reaction when repeatedly exposed to midday sun in summer without protection*				
Mildly tanned/Only freckled or no suntan at all	51	12.6%	63	34.8%
Moderately tanned	175	43.1%	81	44.8%
Very brown and deeply tanned	170	41.9%	36	19.9%
Missing	10	2.5%	1	0.6%
*Total recreational sun exposure (hours)* ^b^				
0–956.7	134	33.0%	45	24.9%
956.7–1387	98	24.1%	46	25.4%
1387–1889.5	91	22.4%	45	24.9%
> = 1889.5	83	20.4%	45	24.9%
*Total sun exposure during regular weekdays/weekends*				
0–362	129	31.8%	45	24.9%
362–584	117	28.8%	46	25.4%
584–851.7	73	18.0%	45	24.9%
> = 851.7	87	21.4%	45	24.9%
*Total sun exposure during vacation in warm and cool months*				
0–18.75	72	17.7%	37	20.4%
18.75–525	68	16.7%	46	25.4%
525–2924.25	112	27.6%	47	26.0%
>2924.25	154	37.9%	51	28.2%
*Region*				
GVRD	244	60.1%	128	70.7%
Capital Regional District	162	39.9%	53	29.3%

^a^ *SD*, standard deviation.

^b^ Total = ∑(Number of years * number of hours per year) for the three types of activities. The number of hours per year is based on the reported number of hours/minutes every time activity is done and the reported number of days engaged in the activity in a year. If the number of days per week is given, this is multiplied by 4.25 weeks per month times 3 months times number of seasons. If the number of days per month is given, this is multiplied by 3 months times number of seasons. If it is number of days per year, then no other multiplier is applied. The number of years is calculated based on the start and end age doing activity (or start and end year doing the activity).

Table 4 presents the results of analyses assessing associations of individual and summed PCB congeners with CMM after adjustment for known melanoma risk factors. Statistically significant trends were observed across quartiles for multiple PCB congeners, including PCBs 138, 153, 170, 180, 183 and 187. For example, with PCB 138, compared to the lowest plasma concentration quartile, OR estimates of 2.3 (95% CI, 1.3–4.1) and 2.4 (95% CI, 1.3–4.5) for the second highest and highest quartiles were observed, respectively. Statistically significantly elevated ORs for CMM were also observed across quartile categories of total PCB levels and total non‐dioxin‐like PCB levels. When comparing the highest to lowest quartile of plasma concentrations, ORs for CMM of 3.1 (95%, CI 1.5–6.3), and 2.6 (1.3–5.3) were observed for total PCBs and total non‐dioxin like PCBs, respectively.

**TABLE 4:** Association between summed and individual PCB congeners with CMM

		Case/Control status							
		Cases *N* = 375^a^		Controls *N* = 171^a^					
PCB congeners	Quartiles (μg/kg of lipids)	*N*	%	*N*	%	OR	95% CI		*p*‐Trend
PCB no. 99^b^	Not detected	280	74.7%	130	73.4%	1.0			0.2
	1.7–107.9	95	25.3%	47	26.6%	1.3	0.8	1.9	
									
PCB no. 105^c^	<0.5	125	33.3%	45	25.4%	1.0			0.9
	0.5 to <0.8	92	24.5%	45	25.4%	0.9	0.5	1.5	
	0.8 to <1.2	84	22.4%	44	24.9%	1.1	0.6	1.9	
	≥1.2	74	19.7%	43	24.3%	0.9	0.5	1.8	
PCB no. 118^d^	<2.5	105	28.0%	45	25.4%	1.0			0.3
	2.5 to <4.4	113	30.1%	45	25.4%	1.7	0.9	3.0	
	4.4 to <6.6	87	23.2%	43	24.3%	1.7	0.9	3.0	
	≥6.6	70	18.7%	44	24.9%	1.7	0.9	3.3	
PCB no. 138^e^	<5.1	94	25.1%	45	25.4%	1.00			0.02
	5.1 to <9	95	25.3%	43	24.3%	1.7	0.9	2.9	
	9 to <13.5	99	26.4%	45	25.4%	2.3	1.3	4.1	
	≥13.5	87	23.2%	44	24.9%	2.4	1.3	4.5	
PCB no. 153^f^	<11.8	88	23.5%	44	24.9%	1.0			0.05
	11.8 to <21.3	125	33.3%	44	24.9%	2.6	1.5	4.6	
	21.3 to <31	85	22.7%	45	25.4%	2.5	1.3	4.7	
	≥31	77	20.5%	44	24.9%	2.7	1.4	5.4	
PCB no. 156^g^	<1.8	103	27.5%	44	24.9%	1.0			0.1
	1.8 to <2.9	102	27.2%	45	25.4%	1.5	0.8	2.7	
	2.9 to <4.5	96	25.6%	45	25.4%	2.2	1.2	4.2	
	≥4.5	74	19.7%	43	24.3%	1.9	0.9	3.8	
PCB no. 170^h^	<3.9	108	28.8%	44	24.9%	1.00			0.04
	3.9 to <6.4	109	29.1%	44	24.9%	1.92	1.0	3.6	
	6.4 to <9.2	65	17.3%	44	24.9%	1.30	0.7	2.6	
	≥9.2	93	24.8%	45	25.4%	2.5	1.21	4.9	
PCB no. 180^i^	<12	109	29.1%	44	24.9%	1.0			0.04
	12 to <19	103	27.5%	44	24.9%	1.9	1.0	3.6	
	19 to <29.5	72	19.2%	44	24.9%	1.7	0.9	3.2	
	≥29.5	91	24.3%	45	25.4%	2.4	1.2	4.9	
PCB no. 183^j^	<0.8	95	25.3%	45	25.4%	1.0			0.01
	0.8 to <1.5	113	30.1%	43	24.3%	1.9	1.1	3.3	
	1.5 to <2.0	68	18.1%	44	24.9%	1.4	0.8	2.7	
	≥2.0	99	26.4%	45	25.4%	2.5	1.3	4.6	
PCB no. 187^k^	<3.1	106	28.3%	43	24.3%	1.0			0.02
	3.1 to <4.8	97	25.9%	46	26.0%	1.5	0.8	2.9	
	4.8 to <7.6	83	22.1%	43	24.3%	2.1	1.1	4.1	
	≥7.6	89	23.7%	45	25.4%	2.5	1.2	5.1	
Total PCB summed^l^	<43.6	93	24.8%	44	24.9%	1.0			0.02
	43.6 to <73.7	109	29.1%	44	24.9%	2.5	1.3	4.6	
	73.7 to <113.4	89	23.7%	44	24.9%	2.8	1.4	5.5	
	≥113.4	84	22.4%	45	25.4%	3.1	1.5	6.3	
Dioxin‐like PCBs^m^	<4.75	100	26.7%	45	25.4%	1.0			0.1
	4.75 to <8.5	122	32.5%	44	24.9%	1.9	1.1	3.4	
	8.5 to <11.9	71	18.9%	44	24.9%	1.4	0.8	2.7	
	≥11.9	82	21.9%	44	24.9%	2.1	1.1	4.1	
Non‐Dioxin‐like PCBs^n^	<39.3	99	26.4%	44	24.9%	1.0			0.02
	39.3 to <65.3	102	27.2%	44	24.9%	2.0	1.1	3.6	
	65.3 to <101.9	91	24.3%	44	24.9%	2.5	1.3	4.8	
	≥101.9	83	22.1%	45	25.4%	2.6	1.3	5.3	

^a^ Total number of cases and controls included in the analysis after excluding individuals with missing covariates.

^b^ Adjusted for age.

^c^ Adjusted for age, education, hair color, skin color, moles, and recreational sun exposure.

^d^ Adjusted for age, education, hair color, moles, sunburn, skin reaction to first exposure to sun without sunscreen, skin reaction to repeated sun exposure without sunscreen, total sun exposure during warm months, sun exposure during vacation in warm regions, and recreational sun exposure.

^e^ Adjusted for age, education, hair color, moles, skin reaction to repeated sun exposure without sunscreen, and total sun exposure during warm months.

^f^ Adjusted for age, education, moles, skin reaction to repeated sun exposure without sunscreen, and sun exposure during vacation in warm regions.

^g^ Adjusted for age, education, moles, skin reaction to first time sun exposure without sunscreen, skin reaction to repeated sun exposure without sunscreen, and recreational sun exposure.

^h^ Adjusted for age, education, hair color, skin color, moles, sunburn, skin reaction to first sun exposure without sunscreen, skin reaction to repeated sun exposure without sunscreen, and recreational sun exposure.

^i^ Adjusted for age, education, moles, sunburn, skin reaction to first sun exposure without sunscreen, skin reaction to repeated sun exposure without sunscreen, total sun exposure during vacation in warm regions, and recreational sun exposure.

^j^ Adjusted for age, education, hair color, moles, skin color, and sun exposure during vacation in warm regions.

^k^ Adjusted for age, education, hair color, skin color, moles, sunburn, skin reaction to repeated sun exposure without sunscreen, total sun exposure during warm months, total sun exposure during vacation in warm region, and recreational sun exposure.

^l^ Adjusted for age, education, skin color, moles, skin reaction to repeated sun exposure, sun exposure during vacation in warm region, and recreational sun exposure.

^m^ Adjusted for age, education, skin color, skin reaction to repeated sun exposure, sun exposure during vacation in warm region, and recreational sun exposure.

^n^ Adjusted for age, education, moles, skin reaction to repeated sun exposure, sun exposure during vacation in warm region, and recreational sun exposure.

^o^ Adjusted for age.

Table 5 provides results of analyses for associations of OC pesticides with CMM risk. Overall, statistically significant trends were observed between CMM and β‐HCH, HCB, Mirex, oxychlordane and trans‐Nonachlor, with OR estimates for the highest versus lowest quartiles ranging from 1.8 (95% CI 1.0–3.3) for HCB to 4.7 (95% CI 2.3–9.7) for oxychlordane.

**TABLE 5:** Association between pesticides and pesticide metabolites with CMM

Pesticide analytes	Quartiles (μg/kg of lipids)	Case/Control status							
		Cases^a^ *N* = 375		Controls^a^ *N* = 171					
		*N*	%	*N*	%	OR	95% CI		*p*‐Trend
β‐HCH^b^	<3.6	90	24.0%	45	25.4%	1.0			0.02
	3.6 to <5.4	93	24.8%	45	25.4%	1.3	0.7	2.3	
	5.4 to <9.7	112	29.9%	44	24.9%	2.1	1.2	3.7	
	≥9.7	80	21.3%	43	24.3%	2.3	1.2	4.4	
									
cis‐Nonachlor^c^	<0.5	94	25.1%	45	25.4%	1.0			0.09
	0.5 to <0.8	116	30.9%	45	25.4%	2.1	1.2	3.7	
	0.8 to <1.3	81	21.6%	43	24.3%	1.8	0.9	3.2	
	≥1.3	84	22.4%	44	24.9%	2.2	1.1	4.2	
									
DDE^d^	<55	112	29.9%	45	25.4%	1.0			0.2
	55 to <102	99	26.4%	43	24.3%	1.3	0.8	2.2	
	102 to <170	74	19.7%	45	25.4%	1.1	0.6	1.9	
	≥170	90	24.0%	44	24.9%	1.5	0.9	2.3	
									
HCB^e^	<7.7	116	30.9%	45	25.4%	1.0			0.02
	7.7 to <9.5	78	20.8%	44	24.9%	0.9	0.5	1.6	
	9.5 to <11.6	83	22.1%	45	25.4%	1.3	0.8	2.3	
	≥11.6	98	26.1%	43	24.3%	1.8	1.0	3.3	
									
Mirex^f^	Not detected	76	20.3%	48	27.1%	1.0			0.03
	0.36 to <1.56	172	45.9%	64	36.2%	2.3	1.4	3.8	
	1.56 to <32.69	127	33.9%	65	36.7%	1.9	1.1	3.3	
									
Oxychlordane^g^	<2.9	81	21.6%	45	25.4%	1.0			<.001
	2.9 to <4.7	105	28.0%	44	24.9%	2.2	1.2	4.1	
	4.7 to <6.8	88	23.5%	45	25.4%	2.5	1.3	4.8	
	≥6.8	101	26.9%	43	24.3%	4.7	2.3	9.7	
									
trans‐Nonachlor^h^	<4.5	94	25.1%	45	25.4%	1.0			<.001
	4.6 to <6.9	90	24.0%	45	25.4%	1.4	0.8	2.5	
	6.9 to <9.9	76	20.3%	44	24.9%	1.7	0.9	3.2	
	≥9.9	115	30.7%	43	24.3%	4.3	2.1	8.5	
									

^a^ Total number of cases and controls included in the analysis after excluding individuals with missing covariates.

^b^ Adjusted for age, education, hair color, skin color, moles, skin reaction to repeated sun exposure without sunscreen, total sun exposure during vacation in warm region, and recreational sun exposure.

^c^ Adjusted for age, education, freckling, skin reaction to first sun exposure without sunscreen, sun skin reaction to repeated sun exposure without sunscreen, total sun exposure during warm months, and recreational sun exposure.

^d^ Adjusted for age, education, hair color, moles, and total sun exposure during vacation in warm region.

^e^ Adjusted for age, education, skin color, and skin reaction to repeated sun exposure without sunscreen.

^f^ Adjusted for age, skin color, and recreational sun exposure.

^g^ Adjusted for age, education, skin color, moles, marital status, skin reaction to repeated sun exposure, and recreational sun exposure.

^h^ Adjusted for age, hair color, moles, skin reaction to repeated sun exposure without sunscreen, total sun exposure during warm months, total sun exposure during vacation in warm region, recreational sun exposure.

## 4 DISCUSSION

In this population‐based case–control study, we observed statistically significant increased odds of CMM in association with several individual PCB congeners and OC pesticides after adjustment for known risk factors including constitutional factors and sunlight exposure. As shown in Table 2, plasma levels of several of these analytes were highly correlated, suggesting that observed associations were not necessarily independent of each other.

Our findings are consistent with data reported in our preliminary case–control study.^4^ Median concentrations of most analytes were slightly lower than in the previous study. This is consistent with previously published observations of declining POP concentrations over time.^19^ Also, in the current study, the population was restricted to females. In general, only a few studies previously assessed sex‐stratified associations of PCBs and melanoma, and no significant sex differences were observed.^7^, ^20^, ^21^ No studies evaluating sex stratified associations between OC pesticides and CMM were identified.

In a recently conducted prospective study among Swedish women, exposure to dietary PCBs (based on food frequency questionnaire) was associated with a four‐fold increased risk of malignant melanoma.^22^ In contrast, recently conducted meta‐analyses do not support the relationship between CMM and exposure to PCBs and OCs.^7^, ^8^, ^23^ While the studies included in the meta‐analyses were mostly from occupational settings with presumably much higher exposure levels, few of the studies involved direct assessment of POP body burdens. Variability in mixtures of PCBs and OCs to which populations are exposed,^6^ as well as heterogeneous study designs (case–control vs. cohort) as well as variability in adjustment for potential confounders (e.g., UV/sun exposure) could potentially explain the mixed associations.^6^, ^7^, ^22^

In addition to chronic inflammation and immunosuppressive effects, it has been shown that PCBs can induce carcinogenesis through prolonged impact on cell receptors (e.g., aryl hydrocarbon receptor [AhR]) leading to cell proliferation, and deregulation of the endocrine system.^5^ While oxidative stress and chronic inflammation have been speculated as mechanisms by which pesticides may increase cancer risk, an actual mechanism has not been identified.^23^, ^24^ PCBs and OC pesticides may also induce carcinogenesis through epigenetic mechanisms. Multiple studies have reported significant associations between DNA methylation and exposure to PCBs and OC pesticides.^25^, ^26^, ^27^, ^28^ Though focused on early life exposures, a recent study found PCBs and OC pesticides to drive sex‐specific changes in DNA methylation.^29^

The large case group and highly detailed covariate data, particularly related to sun susceptibility and sun exposure, are major strengths of this study. Limitations include the use of post‐diagnostic blood samples, in which OC levels may have been impacted by the occurrence of cancer or its treatment. For example, it is known that cancer development and/or its treatment weight loss may lead to increased blood levels of OCs, leading to potential reverse causation.^4^, ^30^ However, as noted earlier, very little weight change between study entry and 2 years before study participation was reported among cases. Furthermore, since among the cases with a reported Breslow thickness value, over two‐thirds (241 out of 359) were diagnosed with thin CMM (Breslow thickness < = 1 mm) (data not provided), it is likely that the majority of cases only received surgical treatment which would have minimal impact on OC levels. Additional limitations include the fact that controls were recruited 1–2 years after cases and the small number of controls available for the analysis. Participation rates in epidemiologic studies, particularly among controls in case–control studies have been declining since 1990.^31^ This has been attributed to an increase in unlisted phone numbers, cell phone usage and screening of calls due to widespread availability of caller identification.^32^, ^33^ While an inadequately representative control population may bias risk estimates,^34^ our findings were consistent with the preliminary study which included a larger number of controls (*n* = 309)^3^ and, in exploratory analyses, associations with known melanoma risk factors were consistent with those previously reported (results not shown). For example, as compared to having a dark skin color, an OR of 2.0 (95% CI 1.3–3.0) for CMM among those with fair skin was observed in our study. This is comparable to the association reported for fair compared to dark skin in a previously conducted meta‐analysis (OR = 1.89; 95% CI, 1.49–2.39).^35^ These findings help reduce concerns about any biases resulting from our control group.

In conclusion, in this study we demonstrated significant associations between various PCB and OC pesticides with CMM risk. Given the highly correlated nature of exposure to PCB and OC analytes, sophisticated analyses that take into account complex mixtures should be considered in future studies.

## CONFLICT OF INTEREST

The authors declare there is no conflict of interest.

## AUTHOR CONTRIBUTIONS

*Formal Analysis, Validation, Visualization, Writing & Original Draft, Writing*–*Review and Editing*, M.D.; *Formal Analysis, Validation, Writing–Review and Editing*, P.B.; *Formal Analysis, Resources, Writing–Review and Editing*, É.G.; *Data Curation, Formal Analysis, Investigation, Software, Writing–Review and Editing*, Z.A.; *Resources, Writing–Review and Editing*, C.C.; *Conceptualization, Funding Acquisition, Methodology, Validation, Writing–Review and Editing*, R.G.; *Conceptualization, Methodology, Validation, Writing–Review and Editing*, J.J.S.; *Data Curation, Funding Acquisition, Investigation, Project Administration, Resources, Supervision, Validation, Writing–Review and Editing*, T.K.L.

## ETHICAL STATEMENT

Ethics was approved by UBC Clinical REB H10‐02669. All participants signed an informed consent.

## Data Availability

The data that support the findings of this study are available on request from the corresponding author. The data are not publicly available due to privacy or ethical restrictions.
